# Anti-hyperalgesic effects of a novel TRPM8 agonist in neuropathic rats: A comparison with topical menthol

**DOI:** 10.1016/j.pain.2014.07.022

**Published:** 2014-10

**Authors:** Ryan Patel, Leonor Gonçalves, Mathew Leveridge, Stephen R. Mack, Alan Hendrick, Nicola L. Brice, Anthony H. Dickenson

**Affiliations:** a Department of Neuroscience, Physiology and Pharmacology, University College London, London, UK; b Takeda Cambridge Ltd., Cambridge, UK

**Keywords:** In vivo electrophysiology, Dorsal horn, TRPM8, Neuropathy, Cold hyperalgesia, Menthol

## Abstract

Menthol has historically been used topically to alleviate various pain conditions. At low concentrations, this non-selective TRPM8 agonist elicits a cooling sensation, however higher concentrations result in cold hyperalgesia in normal subjects and paradoxically analgesia in neuropathic patients. Through behavioural and electrophysiological means, we examined whether this back-translated into a pre-clinical rodent model. Menthol was applied topically to the hind paws of naive and spinal nerve-ligated (SNL) rats. In behavioural assays, menthol did not affect withdrawal thresholds to mechanical stimulation and 10% and 40% menthol rarely sensitised withdrawals to innocuous cooling in naïve rats. However, in SNL rats, 10% and 40% menthol alleviated cold hypersensitivity. This was partly corroborated by in vivo electrophysiological recordings of dorsal horn lamina V/VI neurones. As several studies have implicated TRPM8 in analgesia, we examined whether a novel systemically available TRPM8 agonist, M8-Ag, had more potent anti-hyperalgesic effects than menthol in neuropathic rats. In vitro, M8-Ag activates TRPM8, expressed in HEK293 cells, with an EC50 of 44.97 nM. In vivo, M8-Ag inhibited neuronal responses to innocuous and noxious cooling in SNL rats with no effect in sham-operated rats. This effect was modality selective; M8-Ag did not alter neuronal responses to mechanical, heat or brush stimulation. In addition, M8-Ag attenuated behavioural hypersensitivity to innocuous cooling but not mechanical stimulation. These data suggest that menthol induced hyperalgesia is not consistently replicable in the rat and that the analgesic properties are revealed by injury. Systemic TRPM8 agonists might be beneficial in neuropathy without affecting normal cold sensitivity.

## Summary

An in vivo electrophysiological characterisation of the pro- and anti-nociceptive effects of TRPM8 agonists reveals menthol and a novel TRPM8 agonist have selective inhibitory effects on cold sensitivity in neuropathic rats.

## 1 Introduction

Cold temperatures can be refreshing and relieving, but can also evoke sensations of aching, burning, and pricking. Disturbances in cold sensitivity are a feature of a range of neuropathic conditions including complex regional pain syndrome, chemotherapy-induced neuropathy, and trigeminal neuralgia [2], [20], [44]. The cold mimetic compound menthol has been used to alleviate several painful conditions including musculoskeletal pain [60]. A case study has suggested that high-concentration topical menthol reduces ongoing pain in a patient undergoing chemotherapy [14], but also cold and pinprick hyperalgesia after peripheral neuropathy in a small of group of patients [47], [64]. High concentration menthol applied to uninjured areas has been reported to induce cold hyperalgesia, pinprick hyperalgesia, and in some instances secondary hyperalgesia [8], [27], [65].

The cold and menthol sensitive channel TRPM8 has a multifaceted role in cold sensitivity. Selective fibre blocks suggest that Aδ-fibres are likely responsible for the cooling effects of menthol and cold temperatures, whereas C-fibres are implicated in the paradoxical burning sensation [63], [65]. TRPM8 knockout mice display deficiencies of innocuous cold sensitivity and partly of noxious cold sensitivity [6], [13], [19], [34]. The evidence for TRPA1 being a noxious cold sensor is less compelling [5], [34], [37], and may have a role only in cold hyperalgesia [49]. Both menthol and cooling induced analgesia are dependent on TRPM8 [41], [55], although a TRPM8 negative population of cold sensitive primary afferents are also important. Although cooling is analgesic in both phases of the formalin test in wild-type mice, cooling-induced analgesia is absent in the first phase, but not in the second phase, after genetic ablation of TRPM8 [19]. Through genetic or pharmacological approaches, TRPM8 has been implicated in the development of cold hypersensitivity in several pre-clinical models [13], [17], [35]. Thus, both activating and attenuating the functional roles of TRPM8 channels could be helpful in reducing pain. There are similarities with the TRPV1 channel, a member of the same family of TRP channels, where the agonist, capsaicin, can produce both pain and pain relief and where antagonists are being developed as possible analgesics [48].

Clinical trials involving topical menthol are currently being undertaken for chronic pain conditions including osteoarthritis (clinical trials identifier: NCT01565070), muscle pain (NCT01542827), and chemotherapy-induced neuropathy (NCT01855607). In this study, we have addressed whether the menthol-induced hyperalgesia model in humans is replicable in a rodent model by combining behavioural tests and recordings from WDR neurones in the deep dorsal horn, which integrate low-threshold and noxious mechanical and thermal stimuli. We have also examined whether menthol induces similar analgesic effects in a rodent model of neuropathy, and finally, the systemic effects of a novel TRPM8 agonist, M8-Ag, on cold sensitivity in sham-operated and SNL rats.

## 2 Methods

### 2.1 Animals

Male Sprague-Dawley rats (250–300 g; Biological Services, UCL, UK) were used for behavioural and electrophysiological experiments. Animals were group housed on a 12 h:12 h light–dark cycle; food and water were available ad libitum. All procedures described here were approved by the Home Office, adhered to the Animals (Scientific Procedures) Act 1986, and were designed to reduce numbers and undue suffering in accordance with IASP ethics guidelines [71].

### 2.2 Calcium imaging

TRPM8 agonist activity was determined by measuring changes in intracellular calcium levels using a Ca^2+^-sensitive fluorescent dye. The changes in fluorescent signal were monitored by FLIPR (Molecular Devices, UK). HEK293 cells stably expressing human TRPM8 were seeded in cell culture medium in black, clear-bottom poly-d-lysine-coated 384-well plates 24 hours before the assay (BD Biosciences, Oxford, UK) and grown overnight at 37 °C, 5% CO_2_. On the day of the assay, cell culture media were removed and cells were loaded with Calcium 4 Dye (Molecular Devices, UK) for 1 hour at 37 °C, 5% CO_2_. M8-Ag (4-[5-(4-chlorophenyl)-4-phenyl-4H-1,2,4-triazol-3-yl]morpholine; synthesised in-house) was added to cells and monitored on the FLIPR for 80 seconds. TRPM8-mediated increases in intracellular Ca^2+^ concentration was readily detected upon activation with compound and normalised to a positive control (1 μmol/L icilin, EC_100_). The EC_50_ values were determined from an 8-point half-log concentration–response curve, generated using the average of 2 wells for each data point.

### 2.3 Spinal nerve ligation surgery

Spinal nerve ligation (SNL) surgery was performed as described elsewhere [29]. Rats (125–135 g) were maintained under 2% v/v isoflurane anaesthesia delivered in a 3:2 ratio of nitrous oxide and oxygen. Under aseptic conditions, a paraspinal incision was made and the left tail muscle excised. Part of the L5 transverse process was removed to expose the L5 and L6 spinal nerves, which were then isolated with a glass nerve hook (Ski-Ry Ltd, London, UK) and ligated with a nonabsorbable, 6-0 braided silk thread proximal to the formation of the sciatic nerve. The surrounding skin and muscle were closed with absorbable 3-0 sutures. All rats groomed and gained weight normally in the days that followed after surgery.

### 2.4 Behavioural testing

Behavioural testing of SNL rats was performed 14 days after surgery. Rats were placed inside Perspex chambers on a wire mesh floor and allowed to acclimatise. Using the up–down method [11], 50% withdrawal thresholds were determined with von Frey filaments (Touch-Test; North Coast Medical, Gilroy, CA) proving forces of 1.4 g, 2 g, 4 g, 6 g, 8 g, 10 g, and 15 g. Filaments were applied until they buckled for 5 to 6 seconds. All rats had mechanical hypersensitivity defined as at least a 50% difference in paw withdrawal threshold between ipsilateral and contralateral paws. Cold hypersensitivity was tested by applying a drop of acetone to the plantar surface of the paw using a modified 1-mL syringe. Acetone was applied 5 times, and rats were allowed time to recover between applications. Flinching, licking, shaking, or other directed behaviours were considered positive responses to cold or mechanical stimulation. For the M8-Ag study, only rats with significant cold hypersensitivity were used for analysis, which was defined as 2 or more withdrawals out of 5 on the nerve-injured side (4 of 18 rats were excluded). Rats were then dosed intraperitoneally with vehicle (85% normal saline solution, 10% cremophor (Sigma, UK) 5% dimethylsulfoxide (Sigma, UK), or 30 mg/kg M8-Ag in dose volumes of 2 mL/kg. Behavioural testing was repeated 30, 60, and 90 minutes after dosing. The experimenter was blinded to the treatment during behavioural testing. For the menthol study, 150 μL 1%, 10%, and 40% l-menthol (Alfa Aesar, Heysham, UK) (in absolute ethanol) were cumulatively applied to the entire plantar surface of the left hind paw. Behavioural testing was repeated 15 minutes after each application.

### 2.5 In vivo electrophysiology

In vivo electrophysiology was conducted as previously described [61]. Spinal nerve-ligated and sham-operated rats were used between days 15 and 18 post surgery. Animals were anaesthetised with 3.5% v/v isoflurane delivered in 3:2 ratio of nitrous oxide and oxygen. Once areflexic, a tracheotomy was performed and rats were subsequently maintained on 1.5% v/v isoflurane for the remainder of the experiment. Rats were secured in a stereotaxic frame, and a laminectomy was performed to expose L4 to L5 segments of the spinal cord. Extracellular recordings were made from deep dorsal horn wide dynamic range (WDR) spinal neurones (lamina V/VI) with receptive fields on the glabrous skin of the toes using parylene-coated tungsten electrodes (A-M Systems, Sequim, WA).

Electrical stimulation of WDR neurones was delivered transcutaneously via needles inserted into the receptive field. A train of 16 electrical stimuli (2-millisecond pulses, 0.5 Hz) was applied at 3 times the threshold current for C-fibre activation. Responses evoked by Aβ- (0–20 milliseconds), Aδ- (20–90 milliseconds), and C-fibres (90–350 milliseconds) were separated and quantified on the basis of latency. Neuronal responses occurring after the C-fibre latency band were classed as post-discharge. The input and the wind-up were calculated as Input = (action potentials evoked by first pulse) × total number of pulses (16), wind-up = (total action potentials after 16 train stimulus) − Input. The receptive field was also stimulated using a range of natural stimuli (brush, von Frey filaments [2 g, 8 g, 15 g, 26 g, and 60 g and heat [35 °C, 42 °C, 45 °C, and 48 °C]) applied over a period of 10 seconds per stimulus and the evoked response quantified. The heat stimulus was applied with a constant water jet onto the centre of the receptive field. A 100-μL quantity of acetone and of ethyl chloride (Miller Medical Supplies, UK) were applied as an evaporative innocuous cooling and a noxious cooling stimulus, respectively. Both acetone and ethyl chloride have been previously demonstrated to reliably and reproducibly induce cooling to temperatures comparable to the boundaries for the perception of innocuous and noxious cold. Ethyl chloride is also able to evoke a withdrawal reflex [38]. The neuronal response to room-temperature water was subtracted from acetone and ethyl chloride-evoked responses to control for concomitant mechanical stimulation during application. Receptive fields were determined before electrical stimulation using 8-g and 60-g von Frey filaments. An area was considered part of the receptive field if a response of greater than 50 Hz was obtained. A rest period of 30 seconds between applications was used to avoid sensitisation. Receptive field sizes are expressed as a percentage area of a standardised paw measured using ImageJ (National Institutes of Health, Bethesda, MD).

Data were captured and analysed by a Cambridge Electronic Design 1401 interface coupled to a computer with Spike2 software (Cambridge, UK) with post-stimulus time histogram and rate functions. After 3 consecutive stable baseline responses to natural stimuli (<10% variation; data were averaged to give control values), sham-operated and SNL animals were injected subcutaneously into the contralateral flank with 30 mg/kg M8-Ag. Responses to electrical and natural stimuli were measured 30 minutes post dosing and then every 20 minutes for the next 80 minutes. l-menthol (1%, 10%, and 40%; Alfa Aesar, Heysham, UK) (in absolute ethanol) were cumulatively applied to the receptive field in naive and SNL rats (approximately 20–30 μL). Neuronal responses were tested 15 minutes later. One neurone was characterised per rat.

### 2.6 Statistical analysis

Statistical analyses were performed using SPSS v22 (IBM, Armonk, NY). For in vivo electrophysiology measures, statistical significance was tested using a 1-way or 2-way repeated-measures (RM) analysis of variance (ANOVA), followed by a Bonferroni post hoc test for paired comparisons. Sphericity was tested using Mauchly’s test, and the Greenhouse-Geisser correction was applied if violated. Behavioural time courses and receptive field sizes were tested with the Friedman test, followed by a Wilcoxon post hoc and Bonferroni correction for paired comparisons. Area under the curve (AUC) values were calculated using the trapezoid rule and compared with a 1-way ANOVA followed by a Bonferroni post hoc test.

## 3 Results

### 3.1 Summary of behavioural and neuronal responses in naive, sham, and SNL rats

**Fig. 1:**
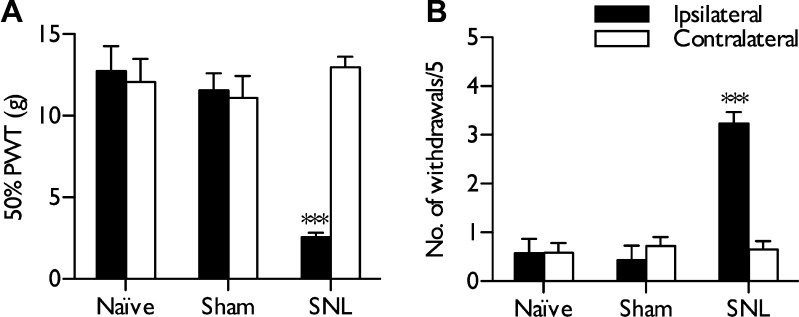
Behavioural hypersensitivity in spinal nerve-ligated (SNL) rats 14 days after surgery. (A) Unilateral ligation of L5 and L6 spinal nerves reduces mechanical withdrawal thresholds and (B) increases responsiveness to acetone-induced innocuous cooling in SNL rats. Sham-operated rats exhibited behavioural sensitivity similar to that in naive rats. Data represent mean ± SEM. ^∗∗∗^*P* < .001. Naive n = 7, sham n = 7, SNL n = 25. PWT, paw withdrawal threshold.

Behavioural sensitivity to mechanical and cooling stimuli was examined 14 days post sham or SNL surgery and in age-matched naive rats. SNL rats displayed guarding behaviour of the injured ipsilateral hind paw, which was absent on the uninjured contralateral side and in sham-operated rats. SNL rats used in this study, but not sham or naive rats, displayed significantly reduced withdrawal thresholds to punctate mechanical stimulation (Wilcoxon test, *P* < .05) (Fig. 1A) and increased withdrawals to acetone-induced innocuous cooling (Wilcoxon test, *P* < .05) (Fig. 1B) compared to contralateral responses.

In vivo electrophysiology was performed to examine the effects of either menthol or M8-Ag on nociceptive processing by wide dynamic range (WDR) spinal neurones in neuropathic and uninjured conditions. Neurones were characterised from depths relating to lamina V/VI in the deep dorsal horn (naive 862 ± 69 μm, sham 704 ± 66 μm, and SNL 691 ± 34 μm) [66], and were confirmed as WDR on the basis of responses to noxious heat, dynamic brushing, and noxious punctate mechanical stimulation. No significant difference was observed in the electrical thresholds for activation of A- or C-fibres or for electrically evoked neuronal responses (1-way ANOVA, *P* > .05). Thermal and mechanical coding of WDR neurones were also similar among naive, sham, and SNL rats (2-way ANOVA, *P* > .05) (Table S1).

### 3.2 Menthol (10% and 40%) alleviates cold hypersensitivity in SNL rats

**Fig. 2:**
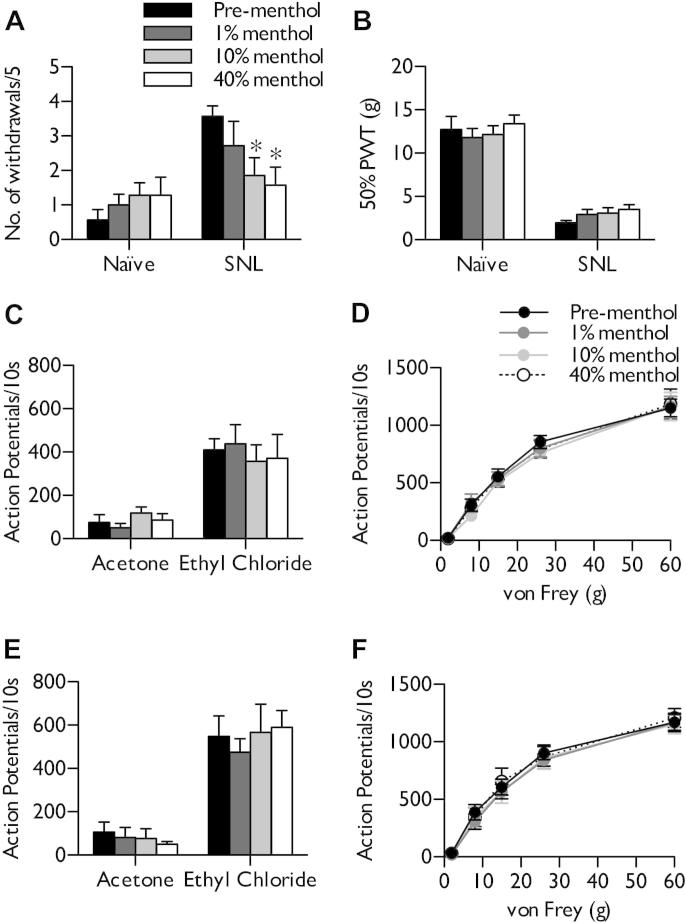
Behavioural and neuronal correlates of mechanical and cold sensitivity after menthol application in naive and spinal nerve-ligated (SNL) rats. (A) Menthol alleviates cold hypersensitivity in SNL rats, with no significant effect in naive rats (n = 7). Corresponding neuronal responses to innocuous and noxious evaporative cooling in (C) naive rats (n = 7) and (D) SNL rats (n = 6). (B) Menthol does not significantly affect mechanical withdrawal thresholds in naive or SNL rats. Neuronal responses to mechanical stimulation in (D) naive and (F) SNL rats corroborate behavioural observations. Data represent mean ± standard error of the mean. ^∗^*P* < .05. PWT, paw withdrawal threshold.

Increasing concentrations of menthol were unilaterally and cumulatively applied to the left hind paw of SNL rats 14 days post surgery and weight/age matched naive rats. Sporadic flinching/shaking behaviours were infrequently observed after application of menthol (naive 2/7, SNL 1/7). In naive rats, no significant sensitisation (2/7 rats sensitised) to acetone-induced innocuous cooling was observed after application of low and high concentrations of menthol (Friedman test, *P* > .05) (Fig. 2A). In contrast, high concentrations of topical menthol reduced acetone-induced withdrawals in SNL rats (Friedman test, *P* < .05, followed by Dunn’s post hoc) (Fig. 2A). Electrophysiological recordings of dorsal horn WDR neurones were made to examine spinal processing of threshold and supra-threshold stimuli. In naive rats, menthol had no overall effect on neuronal responses to acetone-induced innocuous cooling or ethyl chloride-induced noxious cooling (1-way RM ANOVA, *P* > .05) (Fig. 2C). In a further 6 non-acetone-responsive neurones recorded from, only 1 developed cooling sensitivity (data not shown). Only a weak trend towards reduced acetone evoked responses by 40% menthol was observed in SNL rats, with no effect on noxious cold responses (1-way RM ANOVA, *P* > .05) (Fig. 2E). No ongoing neuronal activity was observed after menthol application in either SNL or naive rats.

Menthol had minimal effects on mechanical sensitivity in naive rats (Friedman test, *P* > .05) (Fig. 2B), a feature that was mirrored by a lack of effect on mechanical coding of dorsal horn WDR neurones (2-way RM ANOVA, *P* > .05) (Fig. 2D). In SNL rats, menthol was unable to attenuate significantly mechanical hypersensitivity (Friedman test, *P* > .05) (Fig. 2B), although a subset (3/7) appeared to exhibit a small increase in withdrawal thresholds. Similarly, neuronal responses to mechanical stimulation were also unaltered (2-way RM ANOVA, *P* > .05) (Fig. 2F). Ethanol alone had no effect on neuronal responses (data not shown).

### 3.3 Menthol does not induce sensitisation of deep dorsal horn neurones in naive rats

**Fig. 3:**
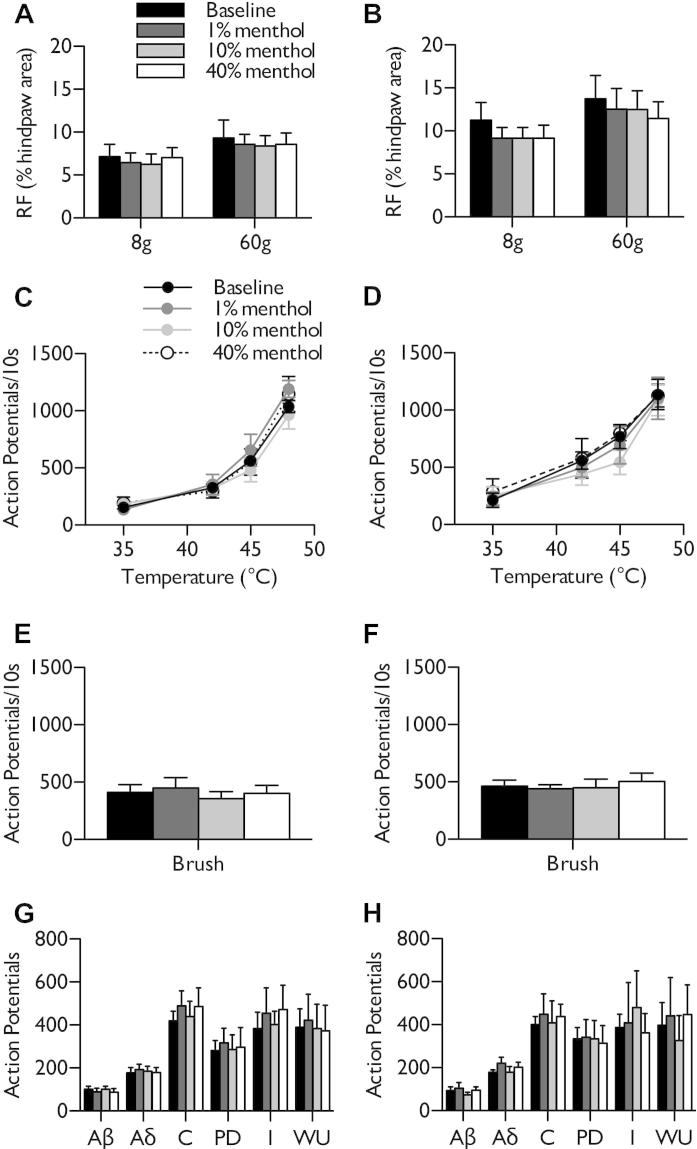
Neuronal responses to natural and electrical stimulation of the receptive field after menthol application in naive and spinal nerve-ligated (SNL) rats. After topical menthol application, no change in receptive field size was observed (A, B) or in heat (C, D), dynamic brush (E, F), or electrically (G, H) evoked responses in naive rats (left panels) or SNL rats (right panels). Data represent mean ± standard error of the mean. Naive n = 7, SNL n = 6. I, input; PD, post-discharge; RF, receptive field; WU, wind-up.

We also examined the effect of menthol on features of neuronal excitability. High-concentration menthol did not induce an expansion of receptive field sizes to innocuous (8 g) or noxious (60 g) punctate mechanical stimulation in naive rats (Friedman test, *P* > .05) (Fig. 3A) and were also unaltered in SNL rats (Friedman test, *P* > .05) (Fig. 3B). In addition, menthol had no influence on neuronal responses to heat stimulation (2-way RM ANOVA, *P* > .05) (Fig. 3C, D) or dynamic brushing (1-way RM ANOVA, *P* > .05) (Fig. 3E, F) in naive or SNL rats. Furthermore, menthol did not sensitise Aδ- and C-fibre-evoked responses or increase the wind-up and post-discharge of WDR neurones in naive rats (1-way RM ANOVA, *P* > .05) (Fig. 3G). After nerve injury, menthol was unable to reduce evoked activity attributed to primary afferent fibres or subsequent wind-up of spinal neurones (1-way RM ANOVA, *P* > .05) (Fig. 3H).

### 3.4 M8-Ag evokes TRPM8-mediated Ca^2+^ currents in vitro

**Fig. 4:**
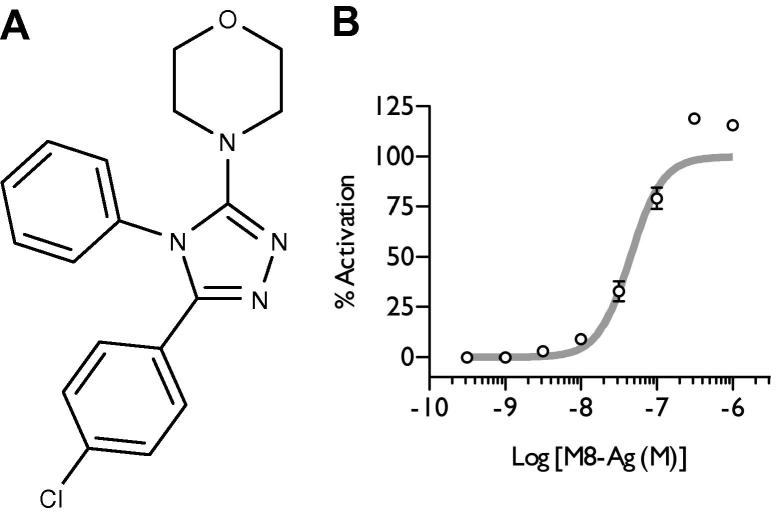
(A) Chemical structure of M8-Ag (4-[5-(4-chlorophenyl)-4-phenyl-4H-1,2,4-triazol-3-yl]morpholine). (B) M8-Ag activates TRPM8 stably expressed in HEK293 cells in a dose-dependent manner. Data are expressed as mean and range of 2 wells.

We next examined the in vitro and in vivo effects of a novel TRPM8 agonist. The ability of M8-Ag (Fig. 4A) to evoke calcium currents was investigated in HEK293 cells stably expressing TRPM8 channels. M8-Ag activated TRPM8 channels in a dose-dependent manner with an EC_50_ of 44.97 ± 1.2 nmol/L (Fig. 4B). M8-Ag also activated TRPA1 but with an EC_50_ of >4000 nmol/L (Fig. S1A). Pharmacokinetic analysis revealed after an intraperitoneal injection of 10 mg/kg M8-Ag in rats, the average peak blood concentration was 4.11 μg/mL (12.06 μmol/L) after 78 minutes with a half-life of 2.6 hours (Fig. S1B).

### 3.5 Neuronal responses to innocuous and noxious cooling attenuated by M8-Ag in SNL rats

**Fig. 5:**
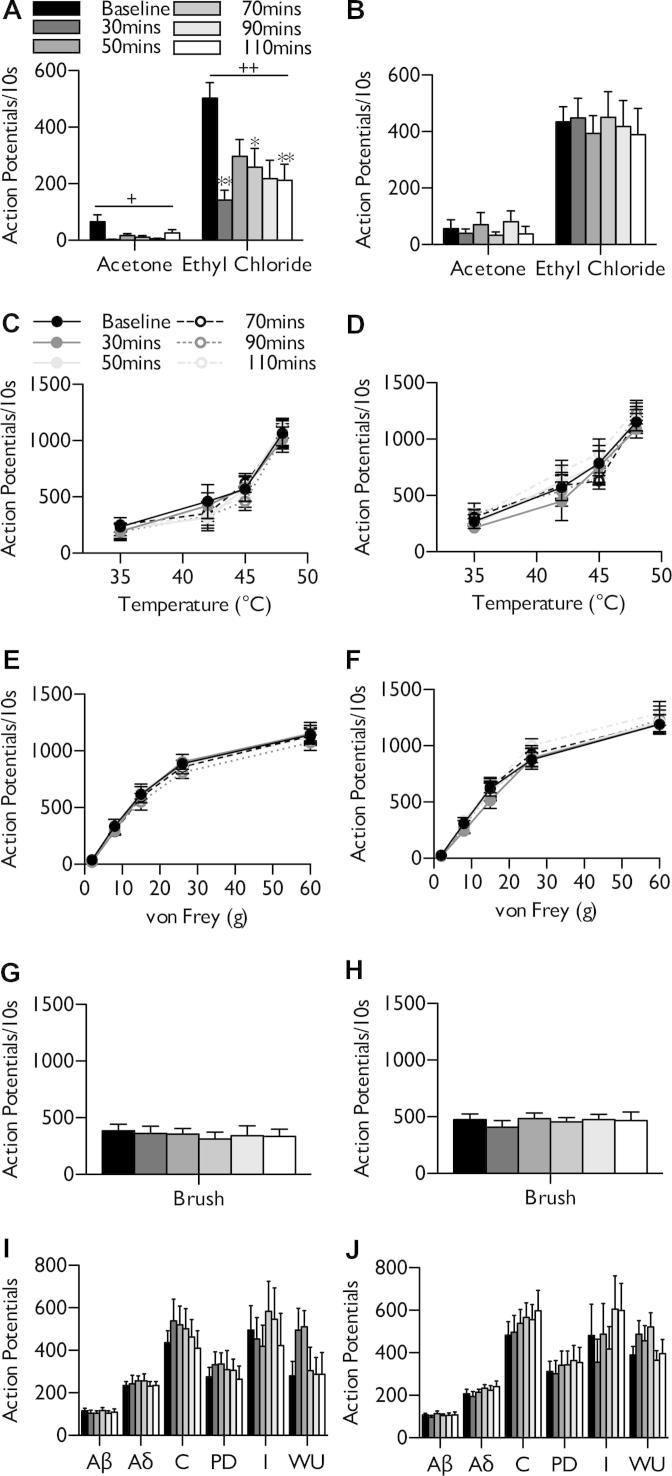
M8-Ag inhibits deep dorsal horn lamina V/VI neuronal responses to innocuous and noxious cold stimulation in spinal nerve-ligated (SNL) rats. After 30 minutes postdosing and then at 20-minute intervals, recordings were made of neuronal responses to cold (A, B), heat (C, D), punctate mechanical (E, F), dynamic brush (G, H), and electrical stimuli (I, J). Left panels, SNL; right panels, sham. (+) represents statistically significant main effect (2-way repeated-measures analysis of variance); asterisks denote significant difference from baseline (Bonferroni post hoc). ^∗^*P* < .05, ^∗∗^*P* < .01, n = 7. I, input; PD, post-discharge; WU, wind-up.

In vivo electrophysiology was performed to examine the effects of systemically dosed M8-Ag on cold sensitive neurones within an integrated system. After obtaining stable baseline neuronal recordings, rats were dosed subcutaneously with 30 mg/kg M8-Ag. In SNL rats, compared to baseline, M8-Ag inhibited neuronal responses to noxious cold stimulation with significantly reduced responses at 30, 70, and 110 minutes after dosing (1-way RM ANOVA, *P* < .01, followed by Bonferroni post hoc test) (Fig. 5A). In addition, M8-Ag decreased overall neuronal responses to innocuous cooling with acetone (1-way RM ANOVA, *P* < .05) (Fig. 5A). These effects of M8-Ag were absent in sham-operated rats (1-way RM ANOVA, *P* > .05) (Fig. 5B). Furthermore, M8-Ag did not alter neuronal responses to heat stimulation (2-way RM ANOVA, *P* > .05) (Fig. 5C, D), punctate mechanical stimulation (2-way RM ANOVA, *P* > .05) (Fig. 5E, F) or dynamic brushing of the receptive field (1-way RM ANOVA, *P* > .05) (Fig. 5G, H) in either sham or SNL rats. Neuronal events evoked by global stimulation of Aβ-, Aδ-, and C-fibres were also unaffected in SNL and sham-operated rats, indicating that activating TRMP8 does not increase overall afferent drive under normal or pathological conditions (1-way RM ANOVA, *P* > .05) (Fig. 5I, J). Vehicle alone in naive rats had no effect on mechanical, cold, heat, or electrically evoked responses (data not shown).

### 3.6 M8-Ag reverses behavioural hypersensitivity to acetone-induced cooling in SNL rats

**Fig. 6:**
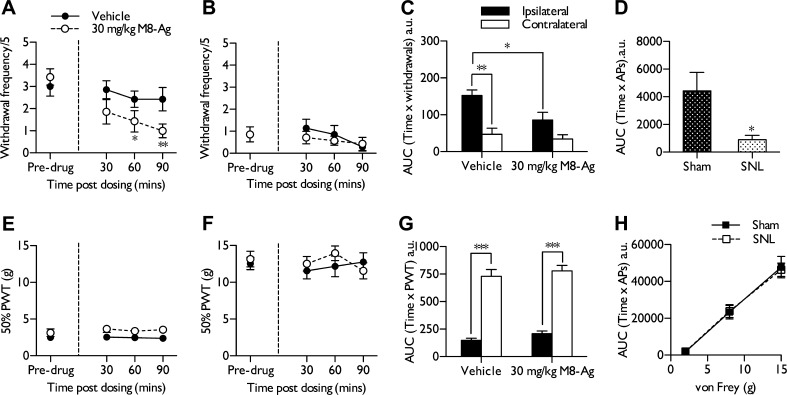
M8-Ag selectively reduces behavioural and neuronal responses to cooling after spinal nerve ligation. (A) M8-Ag, 30 mg/kg, reversed the behavioural response to acetone-induced evaporative cooling on the nerve-injured ipsilateral side compared to pre-drug values (n = 7), whereas vehicle alone had no significant effect (n = 7). (B) Contralateral responses to innocuous cooling were not affected by either treatment. (C) Area under the curve (AUC) analysis confirms a significant attenuation of cold hypersensitivity by M8-Ag compared to vehicle treatment. (D) A decrease in overall neuronal responses to acetone after dosing of M8-Ag was also observed in spinal nerve-ligated (SNL) rats (n = 7) compared to sham-operated rats (n = 7). (E) M8-Ag did not alter mechanical withdrawal thresholds of the injured ipsilateral paw, or (F) the uninjured contralateral side (n = 9). (G) AUC analysis confirms that rats exhibited significant mechanical hypersensitivity, which was not affected by M8-Ag or vehicle treatment. (H) Neuronal responses to mechanical stimulation in SNL rats (n = 7) and sham-operated rats (n = 7) were also unaffected by 30 mg/kg M8-Ag. Data represent mean ± standard error of the mean. ^∗^*P* < .05, ^∗∗^*P* < .01, ^∗∗∗^*P* < .001. APs, action potentials; a.u., arbitrary units; PWT, paw withdrawal threshold.

The ability of M8-Ag to alleviate behavioural signs of cold hypersensitivity was examined in SNL rats 14 days after nerve ligation. Rats were dosed intraperitoneally with either 30 mg/kg M8-Ag or vehicle and tested up to 90 minutes after dosing. The majority of rats dosed with M8-Ag exhibited sporadic ‘wet dog shakes’ up to 1 hour after dosing (7/9). These behaviours were absent in vehicle-treated rats. M8-Ag reduced the behavioural response to innocuous evaporative cooling of the injured paw compared to pre-drug responses (Friedman test *P* < .01*,* followed by Wilcoxon post hoc and Bonferroni correction) (Fig. 6A), whereas the vehicle alone had no significant effect (Friedman test, *P* > .05*)* (Fig. 6A). Contralateral responses to acetone-induced cooling were minimal in comparison and were not affected by M8-Ag (Friedman test, *P* > .05) (Fig. 6B). Overall behavioural signs of cold hypersensitivity are reduced by M8-Ag but not by vehicle treatment (1-way ANOVA *P* < .01, followed by Bonferroni post hoc) (Fig. 6*C*). The behavioural observations correlate with electrophysiological recordings of neuronal responses to acetone. Comparing the effects of 30 mg/kg M8-Ag on total neuronal events in uninjured and SNL rats between 30 and 110 minutes after dosing reveals that cooling-evoked responses were significantly reduced in SNL rats (unpaired Student *t* test, *P* < .05) (Fig. 6D)

M8-Ag did not alter mechanically evoked withdrawals on either the ipsilateral (Fig. 6E) or contralateral (Fig. 6F) side compared to pre-drug values (Friedman test, *P* > .05*).* AUC analysis also confirms no overall effect of either drug or vehicle treatment on mechanical sensitivity (1-way ANOVA, *P* < .001*,* followed by Bonferroni post hoc test) (Fig. 6G). Correspondingly, M8-Ag did not affect mechanical coding of WDR neurones to below threshold and threshold stimuli in SNL or sham-operated rats (2-way ANOVA, *P* > .05) (Fig. 6H).

## 4 Discussion

Several studies support menthol-induced hyperalgesia as a rapidly inducing surrogate model of cold hypersensitivity in human subjects [8], [27], [47], [65]. In addition, menthol also possesses analgesic and counterirritant properties [51], [64]. However, some discrepancies exist between these human studies focused on TRPM8, but also in animal models, as menthol and icilin have been proposed to have complex pro- and anti-nociceptive effects in both uninjured and injured animals [9], [10], [21], [22], [24], [32], [33], [41], [55]. By combining behavioural measures and electrophysiological recordings of lamina V/VI neurones, we have further examined the translational value of this model in uninjured and neuropathic rats (summarised in Table 1 and Table 2).

**Table 1:** Comparison of effects of menthol in naive rats and normal human subjects.

	Rats	Normal human subjects				
	Naive	N = 12	n = 39	n = 10	n = 10	n = 10
Increase in:		Binder et al. (2011)	Hatem et al. (2006)	Wasner et al. (2004)	Namer et al. (2008)	Olsen et al. (2014)
Dynamic mechanical	−	+/−	−	−	+/−	
Punctate mechanical	−	+	−	+		+
Cold	+/−	+	+	+	+	+
Heat	−	+	−	−	−	−
Wind-up	−	−		−		
Secondary hyperalgesia/receptive field	−	+		+/−		

+, Increased response frequently observed, +/−, occasionally observed; −, never/rarely observed; blank, not tested.

**Table 2:** Comparison of effects of menthol in neuropathic rats and neuropathic patients.

	Rats	Neuropathic pain patients			
	SNL	PNP/PHN/CPSP n = 8	CI n = 12	CIN n = 1	PHN n = 1
Decrease in:		Wasner et al. (2008)	Namer et al. (2008)	Colvin et al. (2008)	[16]
Dynamic mechanical	−	−	−		−
Punctate mechanical	+/−	+/−	+/−	+	−
Cold	+	+	+/−		
Heat	−		− (increased)		
Wind-up	−			+	
Receptive field	−				
Ongoing pain				−	+

+, Decreased response frequently observed; +/−, occasionally observed; −, never/rarely observed; blank, not tested; CI, cold injury; CIN, chemotherapy induced neuropathy; CPSP, central post-stroke pain; PHN, post-herpetic neuralgia; PNP, polyneuropathy; spinal nerve-ligated.

We have previously demonstrated that antagonism of the TRPM8 receptor inhibits behavioural and spinal neuronal responses to innocuous and noxious cooling in SNL but not naive rats [53]. Surprisingly, the TRPM8 agonist reported here displayed identical effects. This would imply that TRPM8 is not essential for all forms of cold transduction in the absence of injury in rats, and that the effects of modulators of TRPM8 are dependent on changes induced by the pathophysiological state. Previous studies have demonstrated that menthol sensitises peripheral afferents and trigeminal neurones to cold stimulation [28], [56], [67], and that TRPM8 antagonists can reverse menthol-induced activity in peripheral nerve endings [43]. Menthol does not appear consistently to induce ongoing pain-like behaviours in rats when applied to the paw, mirrored by an absence of ongoing spinal neuronal activity. Menthol may, however, render cool temperatures more aversive in temperature preference assays [32].

Menthol-induced hyperalgesia was designed as a surrogate human model of cold hyperalgesia analogous to the capsaicin model, and some parallels may exist between the two. Hyperexcitability of dorsal horn neurones is considered the neural basis of capsaicin-induced hyperalgesia, in which a prolonged stimulus results in ongoing Aδ- and C-fibre activity and subsequent wind-up, central sensitisation, and secondary hyperalgesia (reviewed by O’Neill et al. [48]). Menthol does not appear to induce comparable changes in neuronal excitability in the rat, which in all likelihood reflects low overall afferent drive from TRPM8+ fibres. WDR neurones did not exhibit potentiated wind-up post menthol application; a similar lack of effect on a perceptual correlate of wind-up was observed in human subjects [8], [65]. All studies in humans have demonstrated an increase in the cold pain threshold (i.e., cold pain experienced at warmer temperatures) after topical menthol with varied effects on other modalities (Table 1). Binder et al. [8] reported incidences of secondary hyperalgesia and pin-prick hyperalgesia as well as heat pain, although over longer time periods than other comparable studies. We did not observe any alterations in evoked neuronal activity by 10% or 40% menthol up to 80 minutes after application (data not shown).

The impact of topical menthol in neuropathic patients has been less extensively examined compared to that in normal subjects. Heterogeneity in the mechanisms of cold allodynia complicates direct comparisons with each other and with pre-clinical models. As a case in point, menthol appears to exacerbate wind-up-like pain in amputees [62], whereas menthol reversed wind-up in a case of chemotherapy-induced neuropathy [14]. The effects of topical menthol were examined in a nerve injury model exhibiting cold hypersensitivity (Table 2). The analgesic properties of menthol in SNL rats appeared restricted to alleviating cooling hypersensitivity, although with no effect on innocuous or noxious cold-evoked neuronal responses. During behavioural characterisations, menthol was applied to the entire plantar surface, whereas with the electrophysiological characterisations, application was restricted to the receptive field of the neurone. Given the paucity of cutaneous TRPM8+ afferents [58], the spatial summation of menthol-evoked activity may be critical in mediating analgesia. Systemic M8-Ag alleviated cold hypersensitivity after SNL and reduced WDR neuronal responses to both innocuous and noxious cooling. The apparent lack of effect of menthol on noxious cold evoked neuronal activity could be attributed to the maximum concentration levels achievable through dermal diffusion. In neuropathic patients, however, the effects of menthol on noxious cold temperatures is difficult to ascertain, as pain is reported at threshold temperatures, and further decreases in temperature cannot be tolerated. These data are consistent with a restoration of spinal gating of cold temperatures at threshold levels by menthol but not of noxious temperatures.

The effects of TRPM8 ligands could result from different consequences of activation of TRPM8 at peripheral levels, and this may be more apparent with topical application. Activation of cutaneous TRPV1 by capsaicin can be sensitising but can also have a desensitising effect due to a combination of acute desensitisation, tachyphylaxis, and withdrawal of epidermal nerve fibres [48]. The mechanism of desensitisation differs between chemical agonists of TRPM8 [36]. Systemic M8-Ag reversal of cold hypersensitivity and hyperalgesia could result from a depolarisation block of TRPM8+ afferents through Ca^2+^-dependent second messenger pathways downregulating TRPM8 activity to prevent calcium excitotoxicity through prolonged activation of channels [15], [54].

There could also be central consequences, either as a result of peripheral TRPM8 targeting or actions on the receptor elsewhere, and these could be more marked with systemic dosing. A disinhibition hypothesis has been proposed to underlie the paradoxical burning associated with small decreases in temperature after nerve damage [50]. If sensitisation of Aδ- and C-fibres underlies the hyperalgesia associated with topical menthol, in an already-sensitised state, rather than exacerbating cold hyperalgesia, one possibility is that activation of TRPM8-expressing afferents with menthol or M8-Ag recruits inhibitory circuits within the dorsal horn, restoring a loss of spinal inhibition [64]. The paradox of a noxious stimulus inducing pain and analgesia according to context is also demonstrable with noxious cold temperatures applied to areas sensitised by capsaicin. The perception of relief could in part be determined by competing aversive and appetitive states converging on descending inhibitory pathways [46]. Functional magnetic resonance imaging analysis suggests that the supraspinal integration of ascending spinal activity and the engagement of descending inhibitory pathways through the periaqueductal grey shapes relief or pain associated with topical menthol or cooling [40], [46], [57]. Although not directly related to the present study, patients with post-stroke pain often have abnormal cold sensitivity, and this is thought to be due to damage to a cool-signaling lateral thalamic pathway that causes a disinhibition of a medial thalamic pathway promoting pain, resulting in the observed burning, cold, ongoing pain and cold allodynia [26]. It is possible that peripheral and then spinal mechanisms via TRPM8 could finally have an impact on this system.

In terms of central mechanisms, TRPM8+ afferents predominantly innervate the superficial dorsal horn, although some afferents terminate in the deeper laminae [18], [58]. Cold temperatures activate a distinct subpopulation of spinothalamic and spinoparabrachial lamina I neurones described as ‘cool’ responsive or ‘polymodal-nociceptive’ [1], [7]. These responses converge onto lamina V/VI neurones, which exhibit graded intensity-dependent responses to decreasing temperatures [31]. Under normal conditions, local glycinergic and GABAergic inhibitory interneurones tonically control inhibitory tone within the dorsal horn and excitability of projection neurones [59]. A substantial cross-inhibition exists within the dorsal horn, whereby low- and high-threshold afferents can moderate central neuronal activity [42], [45]. Group III metabotropic glutamate receptors are highly expressed in the superficial laminae of the dorsal horn, as are group II receptors, which are primarily associated with interneurones in lamina IIi, in proximity to small myelinated fibres, with a large overlap with GABAergic terminals [4], [30], [39]. Both groups of receptors have inhibitory roles within the dorsal horn and are subject to neuroplastic changes after injury [12], [25], [70]. This neuroplasticity is the likely determinant of the reversal of icilin-induced analgesia by group II and III antagonists LY341495 and UBP1112 in neuropathic mice [55]. TRPM8+ afferents also synapse near GABAergic terminals in the superficial dorsal horn [18]. Menthol evokes excitatory postsynaptic potentials in GABAergic interneurones, presumed to activate cold afferents, subsequently gating transmission in projection neurones [69]. Activation of endogenous opioid signalling pathways by menthol has also been implicated in analgesia. Contradictory reports exist of the reversal of icilin and menthol analgesia by naloxone. The discord between these studies could be attributed to the systemic route of administration of menthol [21], [41] as opposed to topically applied icilin [55], as a centrally acting menthol could reduce neuronal excitability through TRPM8-independent, partly opioid mechanisms [52]. Several molecular targets of menthol-mediated analgesia have been proposed, including the cumulative inactivation of sodium channels [23], activation of GABA_A_ currents [68], and inhibition of 5-HT_3_ receptors [3]. The efficacy of menthol is lost in TRPM8 knockout mice, suggesting that these non-TRP mechanisms comprise minor components of anti-hyperalgesia by menthol [41].

### 4.1 Conclusion

In summary, the data presented here suggest that aspects of menthol-induced analgesia are comparable between rats and humans, whereas menthol does not appear to induce central sensitisation as appears to be the case in normal human subjects. Furthermore, M8-Ag attenuated cold behaviours and neuronal responses in neuropathic rats but not in the absence of injury. Thus, overall, an interplay between complex peripheral and central effects appears to underlie the bi-directional effects of TRPM8 ligands, and changes in these functions appear to be driven by damage to peripheral nerves.

## Conflict of interest statement

The authors have no conflicts of interest to declare.

## Appendix A Supplementary data

Supplementary Fig. S1 (A) M8-Ag activates hTRPAI stably expressed in HEK293 cells in a dose-dependent manner. Data are expressed as mean and range of 2 wells. (B) Plasma concentration of M8-Ag after 10 mg/kg intraperitoneal dose (n = 3). Data represent mean ± standard error of the mean.

Supplementary Table S1 Baseline characterisations of deep dorsal horn WDR neurones from naïve, sham and SNL rats. Range of neuronal depths recorded from in parentheses. Data represent mean ± SEM. (APs – action potentials).
