# Supplementary figures and images for: Anti-hyperalgesic effects of a novel TRPM8 agonist in neuropathic rats: A comparison with topical menthol
Source: Pain. 2014 Oct;155(10):2097–107. doi: 10.1016/j.pain.2014.07.022 (PMC4220012; doi:10.1016/j.pain.2014.07.022)

**A**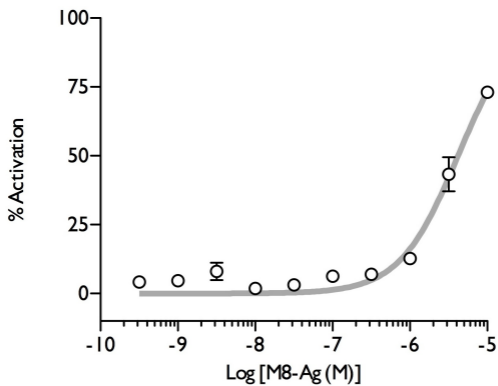**B**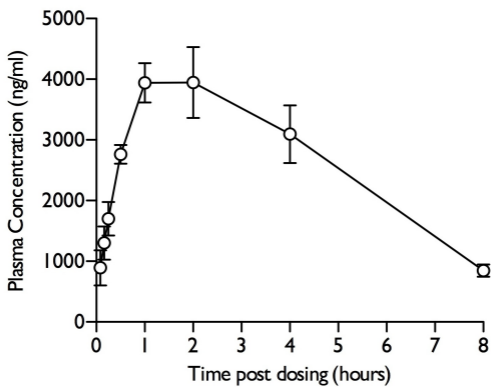

Supplement: Supplementary Fig. S1 — (A) M8-Ag activates hTRPAI stably expressed in HEK293 cells in a dose-dependent manner. Data are expressed as mean and range of 2 wells. (B) Plasma concentration of M8-Ag after 10 mg/kg intraperitoneal dose (n = 3). Data represent mean ± standard error of the mean. [file mmc1.pdf]
