# Supplementary material for: Anti-hyperalgesic effects of a novel TRPM8 agonist in neuropathic rats: A comparison with topical menthol
Source: Pain. 2014 Oct;155(10):2097–107. doi: 10.1016/j.pain.2014.07.022 (PMC4220012; doi:10.1016/j.pain.2014.07.022)
Supplement: Supplementary Table S1 — Baseline characterisations of deep dorsal horn WDR neurones from naïve, sham and SNL rats. Range of neuronal depths recorded from in parentheses. Data represent mean ± SEM. (APs – action potentials). [file mmc2.docx]

|  | **Naïve *n*=7** | | | **Sham *n*=7** | | | **SNL *n*=13** | | |
| --- | --- | --- | --- | --- | --- | --- | --- | --- | --- |
|  |  |  |  |  |  |  |  |  |  |
| **Depth (μm)** | 862 | ± | 68.72 | 704 | ± | 66.22 | 691 | ± | 34.24 |
|  | (620 | - | 1080) | (600 | - | 830) | (470 | - | 1030) |
| **A threshold (mA)** | 0.03 | ± | 0.02 | 0.07 | ± | 0.02 | 0.08 | ± | 0.02 |
| **C threshold (mA)** | 0.29 | ± | 0.11 | 0.43 | ± | 0.16 | 0.33 | ± | 0.10 |
| **Aβ-evoked (APs)** | 102 | ± | 13.75 | 108 | ± | 6.94 | 105 | ± | 10.22 |
| **Aδ-evoked (APs)** | 178 | ± | 23.89 | 207 | ± | 21.94 | 207 | ± | 13.22 |
| **C-evoked (APs)** | 419 | ± | 45.45 | 484 | ± | 62.30 | 418 | ± | 31.65 |
| **Post-discharge (APs)** | 281 | ± | 47.22 | 312 | ± | 48.04 | 305 | ± | 32.08 |
|  |  |  |  |  |  |  |  |  |  |
| **Brush (APs)** | 411 | ± | 65.99 | 474 | ± | 51.06 | 422 | ± | 39.39 |
| **2g (APs)** | 22 | ± | 6.42 | 27 | ± | 8.85 | 36 | ± | 7.89 |
| **8g (APs)** | 305 | ± | 52.29 | 311 | ± | 31.91 | 359 | ± | 38.10 |
| **15g (APs)** | 554 | ± | 48.74 | 620 | ± | 66.70 | 613 | ± | 34.12 |
| **26g (APs)** | 855 | ± | 55.77 | 876 | ± | 63.47 | 894 | ± | 32.20 |
| **60g (APs)** | 1151 | ± | 71.69 | 1192 | ± | 47.53 | 1154 | ± | 40.90 |
|  |  |  |  |  |  |  |  |  |  |
| **35°C (APs)** | 172 | ± | 22.90 | 270 | ± | 61.58 | 225 | ± | 31.18 |
| **42°C (APs)** | 391 | ± | 32.25 | 570 | ± | 114.32 | 501 | ± | 87.31 |
| **45°C (APs)** | 562 | ± | 39.65 | 785 | ± | 109.61 | 652 | ± | 77.07 |
| **48°C (APs)** | 1041 | ± | 144.11 | 1151 | ± | 77.09 | 1095 | ± | 78.42 |
|  |  |  |  |  |  |  |  |  |  |
| **Acetone (APs)** | 75 | ± | 36.32 | 56 | ± | 38.67 | 85 | ± | 24.45 |
| **Ethyl chloride (APs)** | 411 | ± | 49.97 | 434 | ± | 66.84 | 523 | ± | 50.84 |

**Table S1.** Baseline characterisations of deep dorsal horn WDR neurones from naïve, sham and SNL rats. Range of neuronal depths recorded from in parentheses. Data represent mean ± SEM. (APs- action potentials)
